# The role of gut microbiome in mediating the effect of inflammatory bowel disease on hypertension: a two-step, two-sample Mendelian randomization study

**DOI:** 10.3389/fcvm.2024.1396973

**Published:** 2024-10-16

**Authors:** Wei Bao, Yan Zhang, Xiao-Jia Huang, Ning Gu

**Affiliations:** Department of Cardiovascular Disease, Nanjing Hospital of Chinese Medicine Affiliated to Nanjing University of Chinese Medicine, Nanjing, China

**Keywords:** inflammatory bowel disease, gut microbiota, mediator, Mendelian randomization, hypertension

## Abstract

**Objective:**

Investigating the causal connection that exists between inflammatory bowel disease (IBD) and hypertension (HT). To gain a deeper insight into the correlation among IBD, gut microbiota, and HT, we conducted a two-step, two-sample Mendelian randomization study.

**Methods:**

An investigation of genome-wide association study (GWAS) summary-level data was utilized to conduct a two-sample Mendelian randomization (MR) analysis of genetically predicted inflammatory bowel disease: (12,882cases, 21,770controls) on Systolic/Diastolic blood pressure (*N* = 2,564). Subsequently, two-step MR analyses revealed that the relationship between IBD and SBP was partly mediated by *Faecalicatena glycyrrhizinilyticum*. The robustness of the findings was confirmed through several sensitivity assessments.

**Results:**

This MR study showed that increase in genetically predicted IBD was associated with higher risk of genetically predicted SBP (OR: 1.08, 95% CI: 1.01–1.16, *P* < 0.05) and DBP (OR: 1.09, 95% CI: 1.02–1.17, *P* < 0.05), respectively. Inverse variance weighted (IVW) MR analysis also showed that increase in genetically predicted IBD was associated with higher abundance *Faecalicatena glycyrrhizinilyticum* (OR: 1.03, 95% CI: 1.01–1.04, *P* < 0.05), which subsequently associated with increased SBP risk (OR: 1.42, 95% CI: 1.06–1.9, *P* < 0.05). *Faecalicatena glycyrrhizinilyticum* abundance in stool was responsible for mediating 11% of the genetically predicted IBD on SBP.

**Conclusion:**

The research proposed a causal link between Inflammatory Bowel Disease (IBD) and Hypertension (HT), with a little percentage of the impact being influenced by *Faecalicatena glycyrrhizinilyticum* in stool. Mitigating gut microbiome may decrease the heightened risk of hypertension in people with inflammatory bowel disease.

## 1 Introduction

Inflammatory bowel disease (IBD) is associated with gut microbiota dysbiosis, which characterised by means of persistent inflammation affecting the digestive tract with extraintestinal complications that may encompass an array of physiological systems. Globally, extra than 6.8 million sufferers are affected by IBD, the top incidence of IBD takes place between the 2nd and 4th decades of life (1), as a result, these people will be exposed to chronic systemic inflammatory disorders throughout their lives. Patients with IBD are more susceptible to acute vascular events, systemic inflammation and endothelial dysfunction are critically involved in the development and progression of CVDs, emerging evidence has shown that the risk factor is the inflammatory process itself, not any specific ailment, and the contribution of persistent systemic inflammation to atherosclerosis may be the cause of this elevated risk (2). Given the fact that hypertension (HT) is a prevalent and early indication of atherosclerosis, it is logical to expect an increased HT risk in IBD patients. Previous studies have reported a higher prevalence of HT in IBD patients (3, 4). In apparent contrast, another study showed that the prevalence estimates of HT were not significantly different between IBD patients compared with controls. Some studies showed no correlation between IBD and Cardiovascular disease (CVD) (5, 6). Thus, the current data appear inadequate to suggest that HT is associated with increased risk for endothelial dysfunction in IBD patients. Recently, increasing interest has recently focused on the influence of human gut microbiota in HT. Gut microbiota participates in the occurrence and development of hypertension in various ways, existing evidence has proven that gut barrier function, gut microbiota structure, and gut microbial metabolites are key factors involved in the occurrence and development of hypertension (7). Furthermore, microbial sequencing analysis has provided a number of information about the presence of characteristic gut microbiota related to CVDs (8). In addition, other studies also provide evidence of a link between inflammatory bowel disease and the genera of the gut microbiota (9). However, there is currently no data on the genetic effects of gut microbiota in the relationship between IBD and HT.

Mendelian randomization (MR) is an innovative method of analysis in which instrumental variables are genetic variations linked to a potential risk factor to investigate the effect that exposure has on a particular outcome. The random allocation of single nucleotide polymorphisms (SNPs) is governed by Mendel's second rule, making them less susceptible to confounder bias, measurement error, and reverse causality, which are regular in conventional observational research (10, 11). As such, MR has the potential to yield more reliable and effective outcomes that closely resemble those obtained from randomized controlled trials. Moreover, two-step approaches for testing causal mediation using Mendelian randomisation (MR) have been devised. These approaches are significantly less susceptible to the inherent biases found in the conventional multivariable approach (12). In this study, we apply a two-sample, two-step MR design to investigate the associated impacts of IBD on HT and the potential role of gut microbiota.

## 2 Materials and methods

### 2.1 Overall study design

First, in univariate two-sample MR, the bidirectiona relationship between IBD and HT (including SBP and DBP) was analyzed, for the significant causal associations in the univariable MR analysis, the multivariate MR (MVMR) analysis was performed using the MVMR-IVW method, aiming to adjust for potential confounding factors including obstructive sleep apnea, BMI and smoking and drinking. Summary level results of these confounding factors were retrieved from GWAS results based on populations not overlapping with the exposures. In the first step of the two step MR, we conducted a positive MR analysis of the gut microbiota and HT, screened out the GM that were strongly correlated with the disease; in the second step, we used MR to analyze the causal relationship between IBD and the screened mediating GM, and finally derive the *Faecalicatena glycyrrhizinilyticum* abundance in stool that are strongly correlated with the disease, so as to observe the mediating effect of *Faecalicatena glycyrrhizinilyticum* mediating the influence of IBD on HT.

### 2.2 GWAS summary data sources

The summary statistics for GWAS on inflammatory bowel disease were acquired from the International Inflammatory Bowel Disease Genetics Consortium (IIBDGC), including an aggregate number of 34,652 participants of predominantly European ancestry (cases/controls for inflammatory bowel disease: 12,882/21,770) (13). A total of 473 gut microbial taxa were summarized from the genome-wide association study (GWAS) catalog, including 5,959 individuals from FINRISK 2022 cohort (14). Regarding the use of antihypertensive medications, the GWAS summary data for genetic correlations related to Systolic/Diastolic blood pressure obtained from UK Biobank (*N* = 2,564)were adjusted by medication. GWAS data is sourced from several consortia or organizations, ensuring there is no sample overlap. Detailed information about the data sources mentioned above is presented in Supplementary Table S1.

### 2.3 Selection of instrumental variables

Firstly, the instrumental variables chosen to perform analysis must be firmly linked with exposure factors, to guarantee appropriate screening of instrumental variables, SNPs picking up a *p*-value below the locus-wide significance threshold (5 × 10^−8^) were chosen. If there were no notable genome-wide single nucleotide polymorphisms as instrumental variables, SNPs with less than a genome-wide significance level (*P* < 1 × 10^−5^) were engaged as alternative IVs. Secondly, we excluded instrumental variables with *F* values (formula: (*R*^2^/(*R*^2^ − 1)) × ((*N* − *K* − 1)/*K*)) < 10 to guarantee the robustness of the relationship between instrumental variables and exposure factors (15). Thirdly, in order to examine the linkage disequilibrium effect and ensure that the selected instrumental variables satisfy the independence test, we determine the linkage disequilibrium parameter (*R*^2^) of SNP to 0.001 and the genetic distance of 10,000 kb. The palindromic SNPs were removed to prevent the effect of alleles on the outcome. Moreover, SNPs chosen for the exposure and mediator should be distinct, and there were non-overlapping among exposure, mediator and outcome, ensure any effect of the mediator on the outcome is independent of the exposure. To further assess the influence of potential directional pleiotropy, we scanned each of the SNPs used as IVs for their potential secondary phenotypes using the GWAS Catalog (http://www.ebi.ac.uk/gwas, last accessed on August 8, 2024) and performed MR analyses after excluding the SNPs associated with other phenotypes.

### 2.4 Statistical analysis

The most important causal estimate was calculated using the inverse-variance weighted (IVW) approach. This method integrated the Wald ratio of each SNPs to evaluate the result. The heterogeneity of the studies was evaluated using Cochran's *Q* values, *I*^2^ statistics, and the *H* statistics. Other statistical tests including weighted median, MR-Egger, have been used as sensitivity tests. The weighted median technique may provide reliable causal estimates even if half of the weight in the analysis comes from flawed instrumental factors. Horizontal pleiotropy tests were performed by judging the intercept term in MR-Egger regression. If the intercept term was close to 0 (<0.1) and *P* > 0.05, it indicated that there was no evidence of horizontal pleiotropy in the analysis, showing that the results of MR analyses were reliable.

### 2.5 Mediation effects of gut microbiota

**Figure 1:**
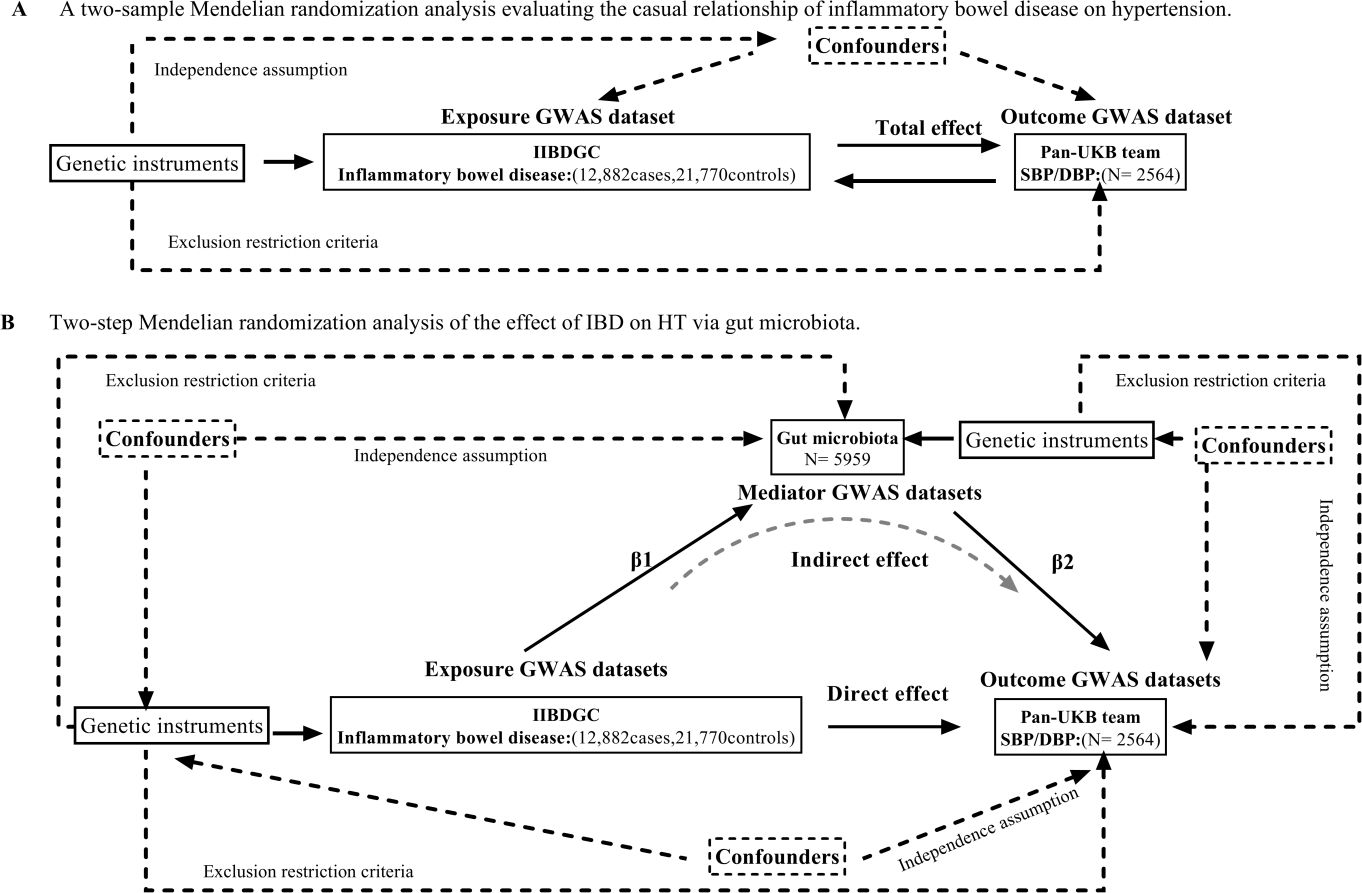
**(A)** Principles of Mendelian Randomization: I) Independence: The genetic variants utilized in the analysis are not associated with any confounders that could potentially influence the relationship between the exposure and the outcome. II) Relevance: The genetic variants selected as instrumental variables have a strong association with the exposure. III) Exclusion Restriction: The genetic variants influence the outcome solely through their effect on the exposure, and not through any alternative pathways; **(B)** The flowchart of two-step MR estimates for the mediation effect of gut microbiota in the causal association between inflammatory bowel disease and hypertension. SBP, systolic blood pressure. DBP, diastolic blood pressure.

Assumptions and design of the a two-sample and mediation Mendelian randomization (MR) analyses. Three key assumptions of MR: (i) the genetic variants are associated with the exposure (the relevance assumption) (ii) genetic instruments are exchangeable with the outcome, across levels of the instrument (the independence assumption) and (iii) the genetic variants do not affect the outcome via any variable other than the exposure (the exclusion restriction criteria) (12). We conducted a mediation study utilizing a two-step Mendelian randomization approach to ascertain the mediation effect of gut microbiota on the associations from IBD to HT outcome (Figure 1). The overall impact of IBD on HT was separated into two components: (1) direct effects of IBD on HT and (2) indirect effects mediated by IBD through the mediator (*β*1*β2 in Figure 1), which *β*1 representing the impact of IBD on gut microbiota, *β*2 representing the consequences of gut microbiota on HT. We estimated the proportion of the total effect mediated by gut microbiota by calculating the ratio of the indirect influence to the overall effect (16). Standard errors for the mediating effects were calculated using the delta method (17).

## 3 Results

### 3.1 Single nucleotide polymorphisms selected in Mendelian randomization

We obtained 63 SNPs in inflammatory bowel disease, which met the widely acknowledged genomewide significance criterion (*P* < 5 × 10^−8^, *r*^2^ < 0.001, kb = 10,000) for exposure. To remove confounding factors, 3 SNPs (rs112694524, rs1873625, rs12446550) strongly associated with hypertension were eliminated. Furthermore, F-statistic estimates revealed the absence of a istrumental weakness bias. (all *F*-statistic > 10). (Supplementary Table S2).

### 3.2 Association of IBD with HT

**Figure 2:**
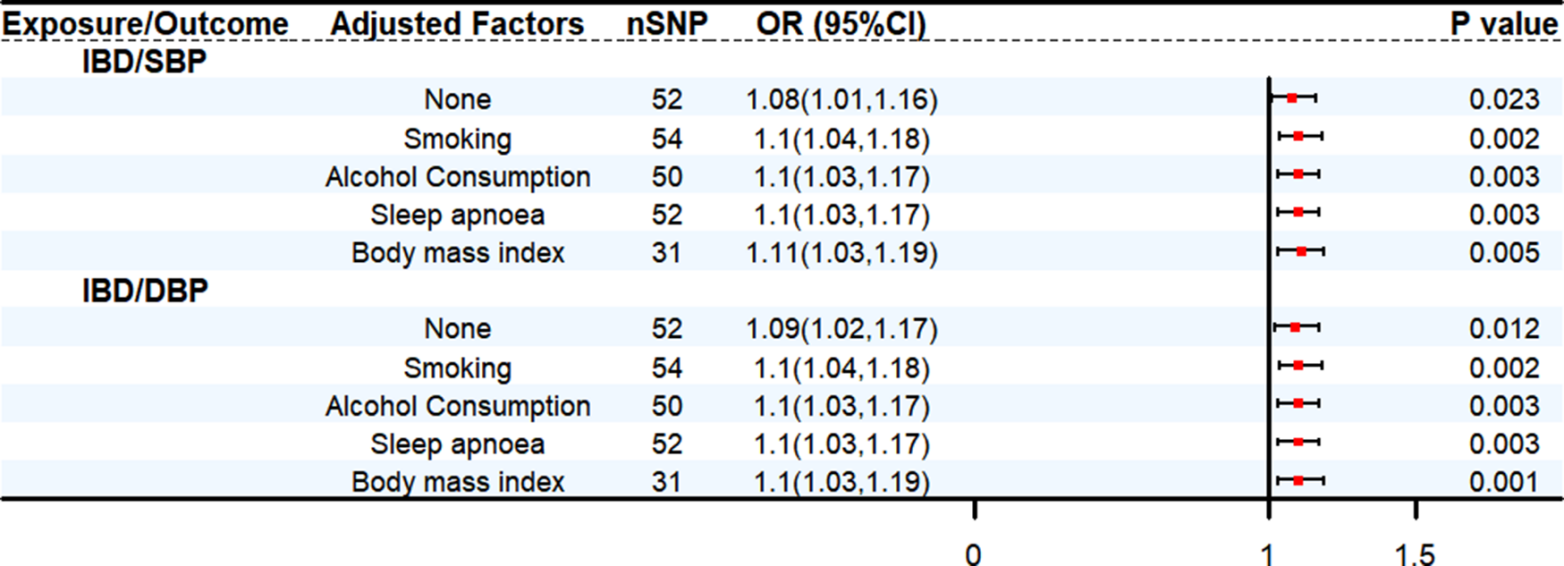
Multivariable MR analysis outcomes. CI, confidence interval.

We conducted bidirectional two-sample MR analyses to assess the relationship between IBD and HT. Mendelian randomization analysis demonstrates that in the primary IVW MR analysis, an increase in genetically predicted IBD was associated with a greater risk of genetically predicted SBP (OR: 1.08, 95% CI: 1.01–1.16, *P* < 0.05) and DBP (OR: 1.09, 95% CI: 1.02–1.17, *P* < 0.05), respectively. Based on the results of reverse analysis, DBP was found positively correlated with the risk of IBD (OR: 1.14, 95% CI: 1.05–1.23, *P* < 0.05) (Supplementary Table S3). The MVMR analysis was conducted to assess the direct effect of IBD on HT with adjustment of multiple other risk factors for HT. The results obtained from the MVMR analysis were consistent with the findings from the univariable MR (Figure 2) (Supplementary Table S4).

### 3.2 Association between gut microbiota and HT

The IVW method discovered 22 causal correlations between gut microbiota characteristics and HT phenotypes (Supplementary Table S5). Specifically, 9 gut microbial taxa were associated with the SBP, 16 gut microbial taxa were associated with DBP.

#### 3.2.1 Systolic blood pressure

**Figure 3:**
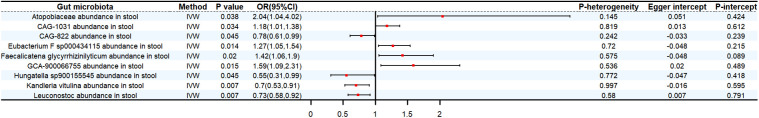
Estimate of causal effect of gut microbiota on SBP.

The genetic forecast *Atopobiaceae* abundance in stool (OR: 2.04, 95% CI: 1.04–4.02, *P* < 0.05), CAG-1031 abundance in stool (OR: 1.18, 95% CI:1.01–1.38, *P* < 0.05), *Eubacterium F sp000434115* abundance in stool (OR: 1.27, 95% CI:1.05–1.54, *P* < 0.05), *Faecalicatena glycyrrhizinilyticum* abundance in stool (OR: 1.42, 95% CI: 1.06–1.9, *P* < 0.05), *GCA-900066755* abundance in stool (OR: 1.59, 95% CI: 1.09–2.31, *P* < 0.05) were positively correlated with SBP. On the contrary, *CAG-822* abundance in stool (OR: 0.78, 95% CI: 0.61–0.99, *P* < 0.05), *Hungatella sp900155545* abundance in stool (OR: 0.55, 95% CI: 0.31–0.99, *P* < 0.05), *Kandleria vitulina* abundance in stool (OR: 0.7, 95% CI: 0.53–0.91, *P* < 0.05), *Leuconostoc* abundance in stool (OR: 0.73, 95% CI: 0.58–0.92, *P* < 0.05) exhibit a protective effect against SBP (Figure 3).

#### 3.2.2 Diastolic blood pressure

**Figure 4:**
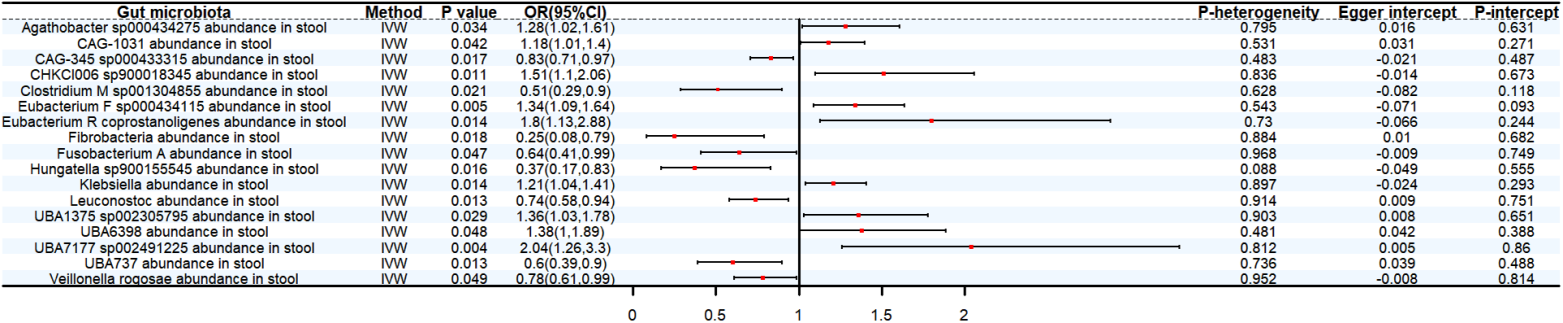
Estimate of causal effect of gut microbiota on DBP.

Genetically predicted *CAG-345 sp000433315* abundance in stool (OR: 0.83, 95% CI: 0.71–0.97, *P* < 0.05), *Clostridium M sp001304855* abundance in stool (OR: 0.51, 95% CI: 0.29–0.9, *P* < 0.05), *Fibrobacteria* abundance in stool (OR: 0.25, 95% CI: 0.08–0.79, *P* < 0.05), *Fusobacterium A* abundance in stool (OR: 0.64, 95% CI: 0.41–0.99, *P* < 0.05), *Leuconostoc* abundance in stool (OR 0.74, 95% CI: 0.58–0.94, *P* < 0.05), *UBA737* abundance in stool (OR: 0.6, 95% CI: 0.39–0.9, *P* < 0.05), *Veillonella rogosae* abundance in stool (OR: 0.78, 95% CI: 0.61–0.99, *P* < 0.05) decreased the risk of DBP. By contrast, 9 bacterial taxa increased the risk of DBP, namely, *Agathobacter sp000434275* abundance in stool (OR: 1.28, 95% CI: 1.02–1.61, *P* < 0.05), *CAG-1031* abundance in stool (OR: 1.18, 95% CI: 1.01–1.4, *P* < 0.05), *CHKCI006 sp900018345* abundance in stool (OR: 1.51, 95% CI: 1.1–2.06, *P* < 0.05), *Eubacterium F sp000434115* abundance in stool (OR: 1.34, 95% CI: 1.09–1.64, *P* < 0.05), *Eubacterium R coprostanoligenes* abundance in stool (OR: 1.8, 95% CI: 1.13–2.88, *P* < 0.05), *Klebsiella* abundance in stool (OR 1.21, 95% CI: 1.04–1.41, *P* < 0.05), *UBA1375 sp002305795* abundance in stool (OR: 1.36, 95% CI: 1.03–1.78, *P* < 0.05), *UBA6398* abundance in stool (OR: 1.38, 95% CI: 1.01–1.89, *P* < 0.05) and *UBA7177 sp002491225* abundance in stool (OR: 2.04, 95% CI: 1.26–3.3, *P* < 0.05) (Figure 4).

### 3.3 Association of IBD with gut microbiota

We then conducted a two-sample Mendelian randomization analysis to examine the relationship between inflammatory bowel disease (IBD) and gut microbiota proved have significant effect with SBP. The IVW methodology identified a positive correlation between genetically predicted IBD and *Faecalicatena glycyrrhizinilyticum* abundance in stool (OR: 1.03, 95% CI: 1.01–1.04. *P* < 0.05) (Supplementary Table S6).

### 3.4 Proportion of the connection between IBD and SBP modified by *Faecalicatena glycyrrhizinilyticum*

**Figure 5:**
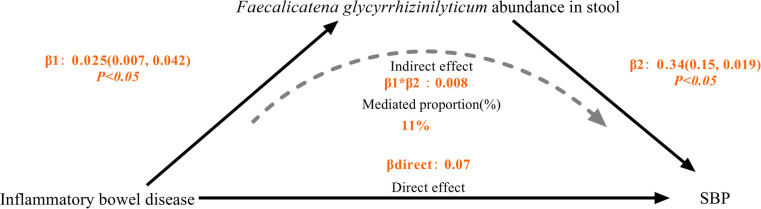
Schematic diagram of the Faecalicatena glycyrrhizinilyticum mediation effect.

After removing gut microbiota not directly impacted by IBD and those without a causal effect on SBP, we took *Faecalicatena glycyrrhizinilyticum* abundance in stool for mediation analysis. We found that IBD was linked to increased abundance of *Faecalicatena glycyrrhizinilyticum* in stool, which thereafter became linked with a increased risk of SBP, the percentage mediated by *Faecalicatena glycyrrhizinilyticum* was 11%. However, the *P* value of the mediation effect was insignificant, and we lacked sufficient evidence to demonstrate that the effect of IBD on SBP was mediated by *Faecalicatena glycyrrhizinilyticum* abundance in stool (Table 1) (Figure 5).

**Table 1:** Mediation MR analysis outcomes.

	Beta	SE	*P* value	Mediated Proportion
Total effect	0.079	0.034	0.023	11%
Direct effect *β*1	0.025	0.008	0.005	
Direct effect *β*2	0.34	0.14	0.019	
Mediation effect	0.008	0.05	0.08	

## 4 Discussion

If gut bacteria play a causal role in the relationship between inflammatory bowel disease and hypertension, this is the first study to investigate this possibility, in the mediation MR analysis, IBD causally increased the abundances of *Faecalicatena glycyrrhizinilyticum* abundance in stool and subsequently associated with increased SBP risk, with a mediated proportion of 11%. Our MR analysis offered proof of a link between hypertension and inflammatory bowel illness, and the role of gut microbiota as mediators.

Our discoveries align with prior research indicating that Inflammatory Bowel Disease (IBD) is linked to an increased risk of excess HT risk, A cross-sectional study from the UK Biobank cohort indicated that patients with IBD had a higher cumulative risk of hypertension compared with general population (18). Previous meta-analysis that drew from 16 trials and a total of 2,029,941 people identified that IBD patients are at higher risk for adverse cardiovascular outcomes, and hypertension accounted for 30% among the most common comorbidities (19). In a large-scale cohort study enrolling 120 consecutive IBD patients, patients with IBD had a considerably greater incidence of white coat hypertension compared to non-IBD controls (20).

Recent data suggests that gut microbial and their impact on intestinal and systemic inflammation are linked to the development and advancement of hypertension (21, 22). *Faecalicatena glycyrrhizinilyticum* abundance in stool was discovered as a mediator in the causative relationship between IBD and HT, the connection between IBD and the microbiota has been confirmed by tests on mice and metagenomic sequencing investigations (22). *Faecalicatena glycyrrhizinilyticum* is Gram-positive, obligate anaerobic, non-spore-forming and rod-shaped bacteria, belonged to cluster XIVa of the genus *Clostridium*. *Clostridium* genus was found in greater abundance in IBD patients, indicating its potential involvement in this disease's pathology (23). *Faecalicatena glycyrrhizinilyticum* was capable of hydrolysing of glycyrrhizin (GL) and generate glycyrrhetic acid (GA) afterwards (24). GA can increase blood pressure by blocking the 11-hydroxysteroid dehydrogenase type 2 and binding to the mineralocorticoid receptor (MR) as an agonist (25). Continuous intake of large amounts of liquorice is a widely known cause of pseudo-hyperaldosteronism leading to hypertension and hypokalemia (26). Our results add to the evidence that increase abundance of *Faecalicatena glycyrrhizinilyticum* was related to lower risk of HT. The strengths of our study include the use of species-level analysis to define gut microbiota taxa, moreover, the GWAS data used in this study come from various populations, which ensured the generalizability of our findings.

### 4.1 Study limitations

Our study has limitations. Firstly, although we strived to minimize pleiotropy, it is impossible to completely eliminate all instances of pleiotropy in MR analysis, unrecognized pathways and confounding factors between the exposure and outcome may still exist, potentially introducing biases into our results. Secondly, our sample size of Hypertension patients is relatively small. Thirdly, The use of genetic IVs indicated that the impacts of the exposures on the results persisted throughout life, which can be in contrast to the current circumstances. Although the indirect effect in the mediation analysis was insignificant, and thus the mediation model was not likely to sustain, our study provides an easily applied framework using both two-step, two-sample Mendelian randomization to investigate potential factors that mediate the causal effect of exposure on the outcome, as well as clarify the extent of the mediation effect (27).

## 5 Conclusion

The present study employed a two-step, two-sample MR approach to ascertain potential causal relationships between HT and IBD, moreover, *Faecalicatena glycyrrhizinilyticum* abundance in stool mediated a minor portion of the observed effect. These results offer support for interventional research investigating the possible function of the intestinal microbiome to mitigate a significant portion of the increased risk of HT in patients with inflammatory bowel disease (IBD). Such investigations could employ a range of therapeutic strategies, such as prebiotics, engineered bacteria (commensal and probiotic), and antibiotics, to regulate the microbiome's abundance (28, 29).

## Data Availability

The original contributions presented in the study are included in the article/Supplementary Material, further inquiries can be directed to the corresponding author.
