# Supplementary material for: The role of gut microbiome in mediating the effect of inflammatory bowel disease on hypertension: a two-step, two-sample Mendelian randomization study
Source: Front Cardiovasc Med. 2024 Oct 16;11:1396973. doi: 10.3389/fcvm.2024.1396973 (PMC11521848; doi:10.3389/fcvm.2024.1396973)
Supplement: Supplementary file 1 [file Datasheet1.pdf]

STROBE-MR checklist of recommended items to address in reports of Mendelian randomization studies[1 2]

Item No.	Section		Checklist item	Page No.	Relevant text from manuscript
1	**TITLE and ABSTRACT**		Indicate Mendelian randomization (MR) as the study's design in the title and/or the abstract if that is a main purpose of the study		Title, Abstract
	**INTRODUCTION**				
2	**Background**		Explain the scientific background and rationale for the reported study. What is the exposure? Is a potential causal relationship between exposure and outcome plausible? Justify why MR is a helpful method to address the study question		Introduction: paragraphs 1-3
3	**Objectives**		State specific objectives clearly, including pre-specified causal hypotheses (if any). State that MR is a method that, under specific assumptions, intends to estimate causal effects		Introduction: paragraphs 4-7
	**METHODS**				
4	**Study design and data sources**		Present key elements of the study design early in the article. Consider including a table listing sources of data for all phases of the study. For each data source contributing to the analysis, describe the following:		Materials and Methods:Study design
		a)	Setting: Describe the study design and the underlying population, if possible. Describe the setting, locations, and relevant dates, including periods of recruitment, exposure, follow-up, and data collection, when available.		Materials and Methods:Data sources,Table S1，Figure 1
		b)	Participants: Give the eligibility criteria, and the sources and methods of selection of participants. Report the sample size, and whether any power or sample size calculations were carried out prior to the main analysis		Materials and Methods:Selection of instrumental variables (IVs) and data harmonization
		c)	Describe measurement, quality control and selection of genetic variants		Materials and Methods:Data sources
		d)	For each exposure, outcome, and other relevant variables, describe methods of assessment and diagnostic criteria for diseases		Materials and Methods:Study design
		e)	Provide details of ethics committee approval and participant informed consent, if relevant		The data analyzed in this secondary study is publicly available from existing, published GWASs and therefore the ethical approval and informed consent have been obtained by all original studies.
5	**Assumptions**		Explicitly state the three core IV assumptions for the main analysis (relevance, independence and exclusion restriction) as well assumptions for any additional or sensitivity analysis		Materials and Methods:Study design and Sensitivity analysis,Figure 1

6	**Statistical methods: main analysis**	Describe statistical methods and statistics used	
	a)	Describe how quantitative variables were handled in the analyses (i.e., scale, units, model)	Detailed information, such as recruitment criteria of population and quality control of genetic data, can be found in the original paper.
	b)	Describe how genetic variants were handled in the analyses and, if applicable, how their weights were selected	
	c)	Describe the MR estimator (e.g. two-stage least squares, Wald ratio) and related statistics. Detail the included covariates and, in case of two-sample MR, whether the same covariate set was used for adjustment in the two samples	
	d)	Explain how missing data were addressed	
	e)	If applicable, indicate how multiple testing was addressed	
7	**Assessment of assumptions**	Describe any methods or prior knowledge used to assess the assumptions or justify their validity	We wsed F-statistics to estimate statistical effectiveness.
8	**Sensitivity analyses and additional analyses**	Describe any sensitivity analyses or additional analyses performed (e.g. comparison of effect estimates from different approaches, independent replication, bias analytic techniques, validation of instruments, simulations)	We used three methods for detecting sensitivity and horizontal pleotropy: weighted median method, MR-Egger and MRPRESSO, F-statistics are used to assess statistical potency, Q-statistics are used to detect heterogeneity,
9	**Software and pre-registration**		
	a)	Name statistical software and package(s), including version and settings used	We performed the analysis by using R software (4.0.2). We used the TwoSample MR package for R to facilitate MR analyses
	b)	State whether the study protocol and details were pre-registered (as well as when and where)	The study was not pre-registered onine
	**RESULTS**		
10	**Descriptive data**		
	a)	Report the numbers of individuals at each stage of included studies and reasons for exclusion. Consider use of a flow diagram	Materials and Methods:Study design,Data sources,Table S1
	b)	Report summary statistics for phenotypic exposure(s), outcome(s), and other relevant variables (e.g. means, SDs, proportions)	Materials and Methods:Study design,Data sources,Table S1-S2

		c)	If the data sources include meta-analyses of previous studies, provide the assessments of heterogeneity across these studies		Detailed information, such as recruitment criteria of population and quality control of genetic data, can be found in the original paper
		d)	For two-sample MR:<br>i. Provide justification of the similarity of the genetic variant-exposure associations between the exposure and outcome samples<br>ii. Provide information on the number of individuals who overlap between the exposure and outcome studies		GWAS data is sourced from several consortia or organizations, ensuring there is no sample overlap. Detailed information about the data sources mentioned above is presented in Table S1.
11	**Main results**				
		a)	Report the associations between genetic variant and exposure, and between genetic variant and outcome, preferably on an interpretable scale		Result:Selection of IVs
		b)	Report MR estimates of the relationship between exposure and outcome, and the measures of uncertainty from the MR analysis, on an interpretable scale, such as odds ratio or relative risk per SD difference		Result:Causal association between IBD with HT,Mediation MR analyses of potential gut microbiota,Table S3-6
		c)	If relevant, consider translating estimates of relative risk into absolute risk for a meaningful time period		N/A
		d)	Consider plots to visualize results (e.g. forest plot, scatterplot of associations between genetic variants and outcome versus between genetic variants and exposure)		Additional visualizations of the results, including scatter plot, forest plot, and leave-one-out plot can be found in Additional file.
12	**Assessment of assumptions**				
		a)	Report the assessment of the validity of the assumptions	4	The evaluation results of the validity of the relevant hypothesis are reported in the full text and several attachments, the statistical power of the instrumental variables in each association is reported, and the results are expressed as F statistics, the heterogeneity of the statistical model was tested with the Q statistic to assess its stability.
		b)	Report any additional statistics (e.g., assessments of heterogeneity across genetic variants, such as I^2, Q statistic or E-value)	4	The I^2 were used to assess the heterogeneity of the causal relationship estimated by each instrumental variable.
13	**Sensitivity analyses and additional analyses**				
		a)	Report any sensitivity analyses to assess the robustness of the main results to violations of the assumptions		Neither horizontal pleiotropy nor heterogeneity (among IVs) was detected at statistically significant

				levels (all P for Egger intercept > 0.05, most of the P for PRESSSO global test > 0.05, and all P for Cochran's Q > 0.05)
		b)	Report results from other sensitivity analyses or additional analyses	Leave-one-out plot can be found in Additional file.
		c)	Report any assessment of direction of causal relationship (e.g., bidirectional MR)	No assessment of the direction of causal association was reported.
		d)	When relevant, report and compare with estimates from non-MR analyses	N/A
		e)	Consider additional plots to visualize results (e.g., leave-one-out analyses)	Leave-one-out analysis: each row represents a twosample MR analysis of exposure on outcome using all of the genome-wide significant SNPs available from Locke et al except for the SNP listed on the y-axis. The point represents the effect size with that SNP removed and the line represents the standard error. Leave-one-out analysis was conducted using MR Base to identify if any individual SNPs were driving the association between exposure and outcome
	**DISCUSSION**			
14	**Key results**		Summarize key results with reference to study objectives	Discussion:paragraph 1
15	**Limitations**		Discuss limitations of the study, taking into account the validity of the IV assumptions, other sources of potential bias, and imprecision. Discuss both direction and magnitude of any potential bias and any efforts to address them	Discussion:paragraph 4
16	**Interpretation**			
		a)	Meaning: Give a cautious overall interpretation of results in the context of their limitations and in comparison with other studies	Discussion:paragraph 2-3
		b)	Mechanism: Discuss underlying biological mechanisms that could drive a potential causal relationship between the investigated exposure and the outcome, and whether the gene-environment equivalence assumption is reasonable. Use causal language carefully, clarifying that IV estimates may provide causal effects only under certain assumptions	
		c)	Clinical relevance: Discuss whether the results have clinical or public policy relevance, and to what extent they inform effect sizes of possible interventions	
17	**Generalizability**		Discuss the generalizability of the study results (a) to other populations, (b) across other exposure periods/timings, and (c) across other levels of exposure	Discussion:paragraph 5

	**OTHER INFORMATION**		
18	**Funding**	Describe sources of funding and the role of funders in the present study and, if applicable, sources of funding for the databases and original study or studies on which the present study is based	This work was partly supported by National Health Commission, Evaluation of endocrine hypertension etiology：　a prospective cohort study （WKZK2022JG0126）
19	**Data and data sharing**	Provide the data used to perform all analyses or report where and how the data can be accessed, and reference these sources in the article. Provide the statistical code needed to reproduce the results in the article, or report whether the code is publicly accessible and if so, where	Data sharing: The data reported in this paper are available by application directly to the IIBDGC, FinnGen and UK Biobank.<br>All code for simulation analyses, applied analyses and example code is available on GitHub (simulations: https:// github. com/ elean orsan derson/ Media tionMR, applied analyses and example code: https:// github. com/ alice rosec arter/ Media tionMR).
20	**Conflicts of Interest**	All authors should declare all potential conflicts of interest	No commercial or financial conflict of interest was identified for this research.

This checklist is copyrighted by the Equator Network under the Creative Commons Attribution 3.0 Unported (CC BY 3.0) license.

1. Skrivankova VW, Richmond RC, Woolf BAR, Yarmolinsky J, Davies NM, Swanson SA, et al. Strengthening the Reporting of Observational Studies in Epidemiology using Mendelian Randomization (STROBE-MR) Statement. JAMA. 2021;under review.

2. Skrivankova VW, Richmond RC, Woolf BAR, Davies NM, Swanson SA, VanderWeele TJ, et al. Strengthening the Reporting of Observational Studies in Epidemiology using Mendelian Randomisation (STROBE-MR): Explanation and Elaboration. BMJ. 2021;375:n2233.
